# Time-Frequency Feature Extraction and Analysis of Inland Waterway Buoy Motion Based on Massive Monitoring Data

**DOI:** 10.3390/s25175237

**Published:** 2025-08-22

**Authors:** Xin Li, Yimei Chen, Lilei Mao, Nini Zhang

**Affiliations:** 1 Department of Port, Waterway and Coastal Engineering, School of Transportation, Southeast University, Nanjing 210096, China; lixintc@seu.edu.cn (X.L.); ninizhang@seu.edu.cn (N.Z.); 2 College of Water Conservancy and Hydropower Engineering, Hohai University, Nanjing 210024, China; maolilei@hhu.edu.cn

**Keywords:** features extraction, wavelet transform, buoy, time series analysis

## Abstract

Sensors are widely used in inland waterway buoys to monitor their position, but the collected data are often affected by noise, outliers, and irregular sampling intervals. To address these challenges, a standardized data processing framework is proposed. Outliers are identified using a hybrid approach combining interquartile range filtering and Isolation Forest algorithm. Interpolation methods are adaptively selected based on time intervals. For short-term gaps, cubic spline interpolation is applied, otherwise, a method that combines dominant periodicity estimation with physical constraints based on power spectral density (PSD) is proposed. An adaptive unscented Kalman filter (AUKF), integrated with the Singer motion model, are applied for denoising, dynamically adjusting to local noise statistics and capturing acceleration dynamics. Afterwards, a set of time-frequency features are extracted, including centrality, directional dispersion, and wavelet transform-based features. Taking the lower Yangtze River as a case study, representative buoys are selected based on dynamic time warping similarity. The features analysis result show that the movement of buoys is closely related to the dynamics dominated by the semi-diurnal tide, and is also affected by runoff and accidents. The method improves the quality and interpretability of buoy motion data, facilitating more robust monitoring and hydrodynamic analysis.

## 1. Introduction

**Figure 1:**
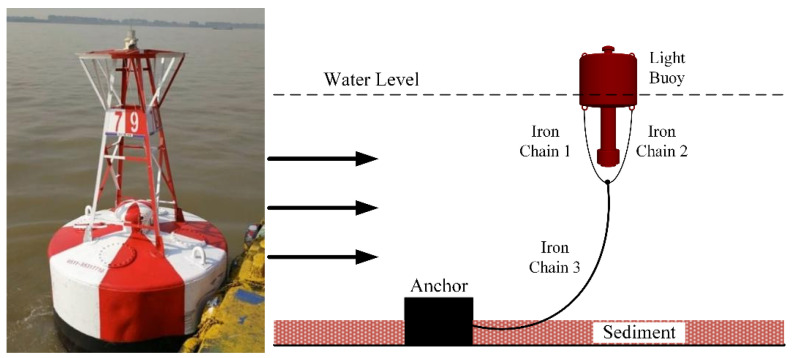
Buoy in the lower Yangtze River.

The Yangtze River, known as the “golden waterway,” traverses the eastern and western economic regions of China, serving as the principal inland water transport corridor and a vital gateway for international trade. The availability of diverse and effective navigation infrastructure, as well as reliable information services, underpins the safety and efficiency of the waterway, which is essential for the smooth functioning of the inland shipping system. Management efficiency has become a key priority for achieving modernization and digital transformation of the shipping governance framework. The construction of the 12.5 m deep water channel downstream of Nanjing has significantly improved navigational conditions, enabled the entry of large ocean-going vessels, and facilitated direct river–sea transportation. However, the complex hydrological and navigational environment of the Yangtze River imposes higher demands on the monitoring and maintenance of navigational infrastructure. As one of the most critical aids to navigation, buoys serve crucial functions including delineating channel boundaries, indicating navigational hazards, conveying route information [1,2], and guiding vessel traffic to ensure navigational safety. In the lower reaches of the Yangtze River, particularly from Nanjing to the estuary, red and black lateral buoys are most commonly deployed to delineate channel boundaries and provide navigational guidance. These buoys are typically moored using single-chain mooring systems fixed to the riverbed [3,4], forming a standard single-point floating structure (as illustrated in Figure 1).

Each channel segment exhibits highly variable sediment transport characteristics and undergoes frequent morphodynamic changes. Meanwhile, each segment features a complex hydrodynamic environment, influenced by both upstream-controlled runoff and the dominant periodic effects of ocean tides [5]. In downstream waterways, single-point mooring buoys are widely deployed due to their cost-effectiveness and ease of installation. However, these buoys operate under challenging hydrodynamic environments and face multiple operational challenges. The direction of tidal currents alternates between day and night, causing the buoy to exhibit periodic fore-and-aft oscillations under the constraint of the mooring chain. During flood tide, the buoy is pushed upstream by the incoming current, while during ebb tide, it drifts downstream. When the tidal current direction shifts abruptly or its velocity diminishes (e.g., during tidal slack), the buoy may experience localized drifting or yaw rotation. Additionally, tidal range variations induce vertical oscillations in water level, resulting in cyclical vertical motion of the buoy. Under the combined effects of wind and waves superimposed with tidal currents, high-frequency vibration or intense swaying may occur over short durations [4]. These frequent oscillations and directional changes accelerate fatigue and wear of the buoy and mooring components, and in extreme cases, may lead to mooring system instability or buoy displacement. Therefore, it is essential to conduct quantitative analyses of buoy motion [6] and extract and classify their dynamic patterns in order to enhance mooring reliability, guide maintenance strategies, and ensure the safe operation of navigational aids.

With the advancement of sensing technology and computational storage capabilities, buoy motion data [7] can now be continuously recorded via high-frequency sensors, generating large-scale spatiotemporal datasets. These data offer a valuable basis for analyzing the movement patterns of buoys and understanding their interaction with hydrodynamic forces. However, due to various limitations—such as fluctuations in river flow, buoy structural characteristics, and variability in communication quality [5]—the raw sensor motion data often contain significant noise, missing values, abrupt outliers, and other anomalies. Such issues compromise the reliability of downstream spatiotemporal analyses. Therefore, prior to any motion pattern investigation, it is necessary to conduct systematic data cleaning and preprocessing, including outlier detection and removal, missing data imputation, trajectory smoothing, and time series resampling, ensuring data completeness, continuity, and physical plausibility. By implementing an automated data cleaning pipeline, overall data quality is enhanced, laying a robust foundation for feature analysis.

Extracting motion features [2,6] from buoy trajectory data is a crucial step for understanding buoy dynamics, evaluating the impact of hydrological environments, and guiding maintenance strategies for navigational aids. As responsive entities within the flow field, buoys reflect the combined effects of local hydrodynamics, vessel-induced disturbances, and mooring stability through their movement patterns. Moreover, due to variations in geographical settings and deployment conditions, buoys in different river segments exhibit significantly different motion characteristics. To capture such variability, this study systematically extracts a range of statistical features—including maximum displacement, mean, standard deviation, quantiles, skewness, and kurtosis—as well as directional indices which capture motion anisotropy. Additionally, several clustering-based spatial indicators are incorporated to reflect similarity. Beyond these time-domain characteristics, this paper obtains the wavelet energy spectrum of each buoy via wavelet transform, and analyzes the frequency spectrum characteristics of the buoy motion. The establishment of a unified feature extraction framework enables quantitative assessment of buoy stability and perturbation intensity, offering strong support for navigation safety monitoring, hydrodynamic anomaly detection, and intelligent buoy management.

Inland waterway buoy trajectories are often contaminated by noise, outliers, and non-uniform sampling. This raises the need for a robust preprocessing and feature extraction framework that can recover and interpret multiscale motion characteristics from noisy and incomplete buoy sensor data. This study makes the following key contributions to the analysis and processing of buoy trajectory data in inland waterways. (1) A novel approach that integrates interquartile range filtering with the isolation forest algorithm for robust outlier detection is proposed. (2) An adaptive interpolation method based on temporal gaps is established, combining dominant periodicity estimation with physical constraints based on power spectral density (PSD). (3) An adaptive unscented Kalman filter, coupled with the Singer motion model, is employed to dynamically adjust to local noise conditions while accurately capturing buoy acceleration dynamics. (4) A comprehensive time-frequency feature framework—including centrality, directional dispersion, and wavelet-based descriptors—is developed to characterize buoy motion, with dynamic time warping applied for similarity analysis and representative buoy selection of different waterway segments, revealing tidal-dominated motion patterns and external influences. The proposed preprocessing and analysis framework can be applied to buoy monitoring data in the lower Yangtze River. It enables the construction of high-quality, spatiotemporally consistent trajectory datasets and reveals key motion characteristics. These outcomes support the development of smart buoy monitoring systems and contribute to improved reliability, navigation safety, and service quality in inland waterway transport.

The remainder of this paper is organized as follows. Section 2 provides a literature review of the research related to time series data process and feature extraction of buoys. Section 3 presents a detailed introduction to outlier removal, interpolation, and noise reduction. Section 4 takes various segments of the lower Yangtze River as examples, extracting and analyzing features to discover movement patterns of different buoys. Finally, Section 5 summarizes the conclusions.

## 2. Literature Review

Buoy trajectory data, influenced by hydrodynamic fluctuations, intrinsic buoy characteristics, sensor interference, and communication faults, often exhibit non-stationarity, non-linearity, and multi-scale behaviors. Such data frequently contain outliers and missing values, necessitating comprehensive preprocessing before spatiotemporal analysis. Outlier detection methods generally fall into three categories: statistical, machine learning, and deep learning approaches [8]. In the statistical domain, the interquartile range (IQR) method is commonly used. It identifies outliers as those falling outside the lower and upper bounds derived from quartile statistics. While simple and interpretable, IQR is more suited to dispersed datasets and can lead to over-flagging in high-variance time series. Among machine learning techniques, algorithms such as K-Nearest Neighbors (KNN), Random Forest, Support Vector Machines (SVM), and Isolation Forest (IF) have gained popularity. Notably, Isolation Forest, proposed by [9], constructs random decision trees to isolate anomalies. Its computational efficiency and robustness in high-dimensional data make it well-suited for large-scale time series anomaly detection. Deep learning-based anomaly detection, particularly for high-dimensional, complex, and dependent time series, is also gaining traction [8,10]. However, these models require large datasets and high computational costs. Given the volatility and data limitations in our context, this study adopts a hybrid method combining the IQR approach and Isolation Forest to balance accuracy and efficiency.

Regarding missing value imputation, maintaining consistent temporal resolution and structural coherence are essential [11]. Interpolation methods such as linear interpolation, quadratic splines, and cubic splines are widely used. Cubic spline interpolation preserves the continuity of both first- and second-order derivatives, making it ideal for smooth and accurate filling of missing values. However, traditional interpolation techniques often neglect the non-uniform sampling and periodic behaviors of real-world time series [12]. Consequently, this study proposes a phase-wise imputation strategy based on temporal gap identification and periodic pattern recognition to improve interpolation accuracy.

Noise is another key challenge in time series analysis. Traditional statistical smoothing methods lack adaptability to dynamic systems. Frequency-domain techniques (e.g., Fourier or wavelet transforms) [13] struggle with non-stationary signals. Deep learning models, though powerful, are computationally intensive and sensitive to data noise. Recursive filtering methods, particularly the Kalman filter (KF) [2] and its variants such as extended KF (EKF), unscented KF (UKF), and particle filters (PF) [14,15], are widely used due to their adaptability to dynamic state estimation. Kalman filters have been successfully applied in buoy data denoising and navigation systems [16,17], but their reliance on linear and Gaussian assumptions limits performance under nonlinear and non-Gaussian conditions. Particle filters offer flexibility by approximating the posterior distribution via Monte Carlo sampling, making them suitable for complex systems. However, PF [18] suffers from particle degeneracy and computational cost. The cubature particle filter (CPF), which combines UKF with PF, has been proposed to improve particle effectiveness [19]. Despite CPF’s advantages, its computational complexity limits real-time applicability. In this study, we adopt the unscented Kalman filter (UKF) due to its better computational efficiency, and further enhance it via an adaptive mechanism (AUKF) that adjusts covariance matrices based on observation error statistics. This allows for dynamic noise estimation without prior distribution assumptions, making it suitable for buoy trajectories with nonlinear and irregular noise profiles. To better model buoy motion dynamics, the Singer model is incorporated. It captures acceleration behavior during motion transitions and is often used in maneuvering target tracking. By modeling autocorrelated acceleration, the Singer model reflects both inertial and disturbance properties in trajectory evolution. When combined with AUKF, this hybrid approach enables robust and physically informed trajectory smoothing, improving accuracy and filter stability in complex riverine environments.

In terms of feature extraction, Han et al. [20] proposed an ensemble learning framework including several features, such as statistical, temporal, spectral, and wavelet features. Time-domain features are statistical quantities extracted directly from raw time series data. They quickly reveal the overall trends and fluctuation amplitudes of buoy motion and describe periodic or stable motion states. While Cheng et al. [21] proposed a neural network framework emphasizing the importance of frequency-domain features in sea state estimation. They used the fast Fourier transform (FFT) to convert time-domain signals into frequency-domain representations, and then used the CNN to extract effective frequency features. Some studies also have explored frequency-domain characteristics. Frequency-based methods, such as empirical mode decomposition (EMD) [22,23], wavelet analysis [24,25], and singular spectrum decomposition [26], have been used to extract oscillatory components of different frequencies, which can be used to model different time series separately [27]. Peng [28] used wavelet packet decomposition to extract fault signal features from wave sensor data. Based on the statistical results, standardized data preprocessing and wavelet transform algorithms were applied to wave-related variables. Pan et al. [29] investigated the time-frequency characteristics of the dynamic responses induced by the freak wave and the differences compared with responses under random waves. Chakraborty et al. [30] discussed the time-frequency analysis of collected wave signals using the short-term Fourier transform (STFT). This paper considers the feature extraction in both time and frequency domains to analyze the motion characteristics of different buoys. Despite the progress made in extracting statistical, spectral, and time-frequency features from marine signals, existing studies often focus on open-sea wave sensors or stationary structures, with limited attention to inland waterway buoys, which exhibit nonstationary, anisotropic, and locally driven motion patterns. Moreover, many approaches lack unified preprocessing pipelines, resulting in inconsistencies in temporal resolution, noise interference, and missing data handling. Current feature extraction frameworks also tend to consider time- and frequency-domain indicators separately, without integrating them to capture multiscale dynamic responses from various hydrodynamic drivers such as tides, runoff, and sudden impacts. To address these gaps, this study proposes a complete data preprocessing and feature extraction framework tailored to inland waterway buoy trajectories. It incorporates robust anomaly detection, adaptive interpolation, and physically informed denoising, followed by the construction of a multiscale time-frequency feature system. This enables systematic identification of abrupt changes, dominant oscillation modes, and tidal/runoff influence, thereby enhancing the interpretability and reliability of buoy motion characterization.

## 3. Methodology

### 3.1. Study Area

**Figure 2:**
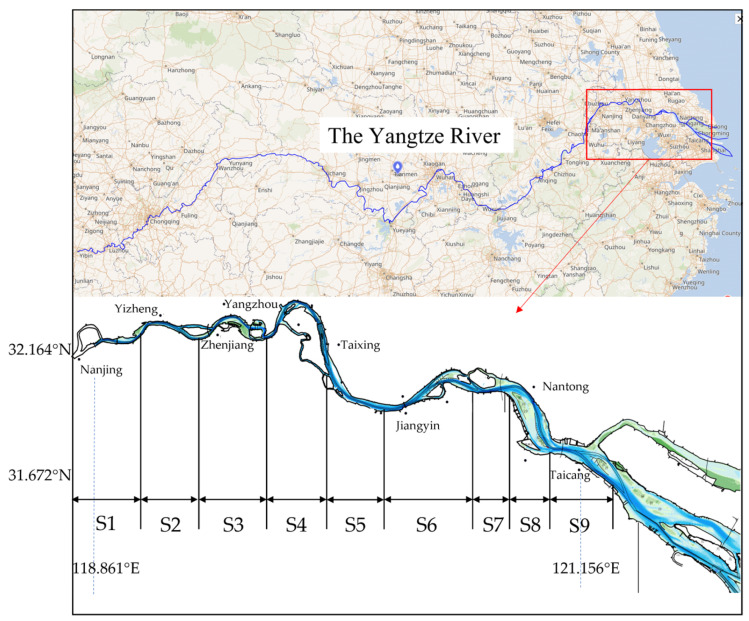
Study area.

The river reach stretching from the Nanjing Yangtze River Bridge downstream to the estuary at Chongming Island represents a typical tide-affected section of the Yangtze River. It is one of the most important navigable main channels in the lower Yangtze River and serves as a critical transitional zone between inland waterways and coastal waters (see Figure 2 for the study area).

The study area spans from Nanjing to Taicang, encompassing a number of morphologically diverse waterways. From upstream to downstream, the major sub-reaches are denoted in Table 1.

### 3.2. Collected Data

Single-point mooring buoys are critical facilities for both navigation marking and hydrological monitoring in the lower reaches of the Yangtze River, and have been widely deployed throughout the region. To investigate the dynamic responses of buoys under varying hydrodynamic conditions, it is necessary to collect time series data of buoy sensors. The sensor data used in this study were obtained from the hydrographic monitoring system operated by the Nanjing Navigation Bureau of the Yangtze River. The selected data cover the above river reach, during the dry season of the Yangtze River, from 00:00 on 5 January 2021, to 00:00 on 25 April 2021. A total of 458 buoys were initially recorded, distributed across straight channels, narrow bends, bridge zones, and bifurcated reaches. To ensure data reliability and avoid distortions caused by missing data in subsequent analyses, buoys with incomplete historical records were excluded. As a result, 295 buoys were retained, yielding approximately 850,000 timestamped position records in total.

To facilitate spatial analysis, the recorded geographic coordinates (latitude and longitude) were projected into a Cartesian coordinate system using the Mercator projection. The projection system follows the China Geodetic Coordinate System 2000 (CGCS2000) with Gauss–Krüger projection, central meridian set at 120° E, and standard offset values applied: +500,000 m (East) and +0 m (North). The vertical datum is based on the 1985 National Height Datum, and the depth reference is set to the navigational chart datum.

### 3.3. Manual Displacement Identification

The collected dataset includes instances of abrupt changes in buoy position caused by repositioning behavior, which refers to the manual relocation of buoys or system-driven adjustments due to unsatisfactory performance or maintenance of the original deployment sites. To filter out such abnormal data, this study adopts a hybrid approach that combines a sliding window-based mean absolute variation rate detection method with a mooring chain length constraint.

First, based on the positional data p(t)=(x(t),y(t)) of each buoy at time t, the displacement between consecutive time points $∆P_{t}=||p(t)-p(t-∆t)||$ is calculated. Using the time interval ∆t, the rate of change of position (i.e., velocity magnitude) is computed as:

(1) $$v(t)=\frac{\left||p(t)-p(t-∆t)|\right|}{∆t}$$

To quantify the movement intensity, we consider the absolute variation rate |v(t)|, and apply a moving average filter with a window length w=3 h to detect abnormal movements.


(2) $$\tilde{v}(t)=\frac{1}{2k+1}\sum_{i=-k}^{k}\left|v(t+i)\right|,\mathrm{where}\;k=\frac{w-1}{2}$$

An anomaly threshold θ as the 98th percentile of the distribution of $\tilde{v}(t)$. Time points satisfying $\tilde{v}(t)>\theta$ within local temporal windows are flagged as potential anomalies, indicating unusual buoy motion beyond normal oscillatory behavior.

**Figure 3:**
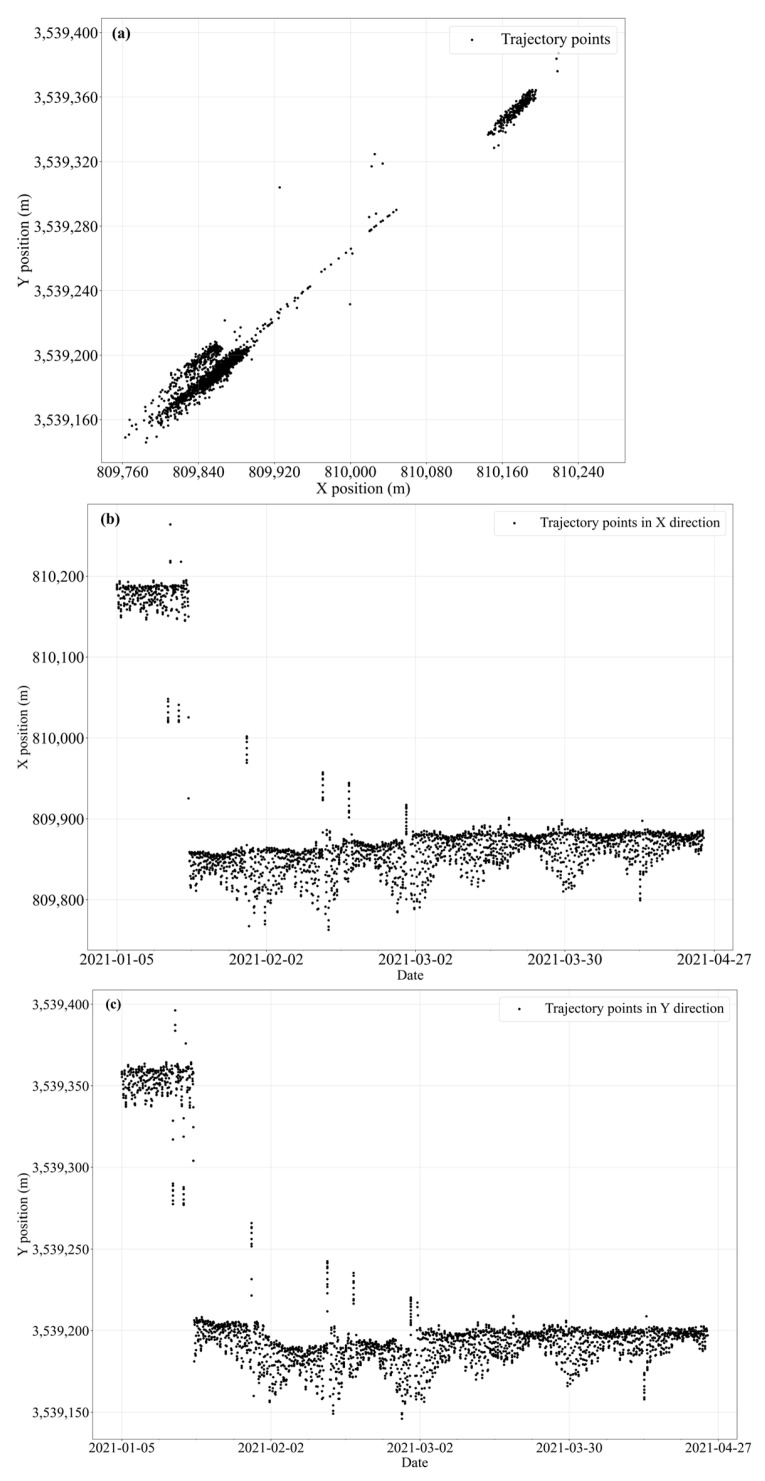
Distribution of the #60 red buoy; (**a**) is the change in the buoy position, (**b**) is the change in the x-coordinate, and (**c**) is the change in the y-coordinate.

Considering the physical constraint imposed by the mooring chain, the buoy’s movement is confined to a circular area of radius r=L, where L is the mooring chain length. $D(t_{1},t_{2})$ is defined as the cumulative displacement over a short time span [$t_{1},t_{2}$]. If:

(3) $$D(t_{1},t_{2})=\sum_{t=t_{1}}^{t_{2}}∆P_{t}>L,\exists t\in [t_{1},t_{2}],\tilde{v}(t)>\theta$$

then the movement is classified as non-natural and attributed to a manual repositioning event. Through the combined application of the sliding window variation rate and the mooring length constraint, a subset of manually relocated buoys was successfully identified and excluded from subsequent analyses. Taking the #60 red buoy in S6 as an example, the spatial distribution of its movement points is visualized, with the horizontal and vertical axes representing the projected coordinates, as shown in Figure 3.

From the figure, it is evident that the buoy exhibits a distinct step-like pattern in both the X and Y directions, indicating abrupt positional shifts. Spatially, the buoy’s trajectory points are clustered into two separate regions, which are clearly distant from each other. This distribution pattern suggests that the buoy underwent manual repositioning behavior during the observation period. Consequently, buoys with such evident repositioning behaviors should be excluded from subsequent analyses of buoy motion patterns and feature extraction.

### 3.4. Joint Detection Method for Trajectory Outliers

To effectively identify and eliminate abnormal trajectory points from buoy movement data, this study adopts a hybrid anomaly detection method that combines the IQR technique and the Isolation Forest algorithm. The IQR method [31] is a classical statistical approach for outlier detection. It leverages the distributional characteristics of the data to construct a robust threshold for identifying anomalies. Given a time series of a specific feature variable $X=\{x_{1},\ldots ,\;x_{n}\}$, the first (Q1) and third (Q3) quartiles correspond to the 25th and 75th percentiles [32], respectively. The interquartile range is then calculated as IQR=Q3−Q1. A point $x_{i}$ is considered an outlier if it satisfies the following condition:

(4) $$x_{i}<Q1-k\cdot IQR\;||\;x_{i}>Q3+k\cdot IQR$$

where *k* is typically set to 1.5 or 3, indicating moderate or severe outliers [32], respectively. The IQR method has the advantages of being non-parametric, computationally efficient, and sensitive to abrupt anomalies, such as GPS drift or positional jumps. However, it has limitations when dealing with multivariate trajectory data, where inter-variable relationships are complex and nonlinear.

**Figure 4:**
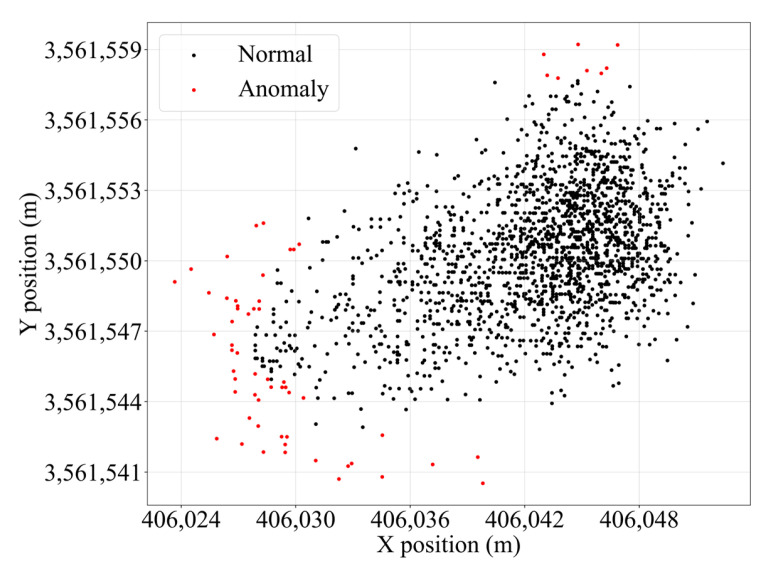
Anomaly point removal of the #131 red buoy.

To overcome this limitation, the Isolation Forest algorithm is introduced, which is well-suited for high-dimensional and nonlinear data structures. Isolation Forest is an ensemble-based unsupervised anomaly detection method built on the principle of random partitioning. Unlike density-based methods that rely on estimating data distributions, Isolation Forest directly isolates observations by randomly partitioning the data space. It constructs multiple binary trees (termed isolation trees) by recursively and randomly selecting a feature and a split value. Anomalous points, which are easier to isolate, tend to have shorter average path lengths across trees. For the dataset $X=\{x_{1},\ldots ,\;x_{n}\}$, the anomaly score s  for a given point $x_{i}$ is defined as:

(5) $$s(x_{i},n)=2^{-\frac{h(x_{i})}{c(n)}}$$

where $h(x_{i})$ is the average path length of $x_{i}$ over all trees, and c(n) is the average path length for unsuccessful searches in a binary search tree, approximated as:

(6) $$c(n)=2H(n-1)-\frac{2(n-1)}{n}$$

with H(i) denoting the i-th harmonic number. A score $s(x_{i})$ close to 1 implies a high probability of being anomalous, whereas scores near 0.5 indicate normal behavior. This mechanism makes Isolation Forest highly efficient for large-scale, high-dimensional datasets without requiring assumptions about the underlying distribution. For example, the trajectory data of the #131 red buoy in S1 is used to illustrate the effectiveness of this combined anomaly filtering approach. The results of anomaly point removal are shown in Figure 4.

### 3.5. Missing Value Processing Methods

Due to potential issues such as non-integer sampling timestamps, data loss, and transmission errors, the recorded buoy position sequences may exhibit irregular time intervals. A dual-mode interpolation strategy based on the length of missing intervals is proposed. For gaps shorter than 6 h, cubic spline interpolation is applied to reconstruct the missing values. For long-term gaps (≥6 h), the method switches to spectral analysis-guided interpolation, leveraging the dominant periodicity inherent in the cleaned trajectory. This method facilitates fast interpolation, combining spectral features and physical constraints to form a dataset with uniform time intervals.

#### 3.5.1. Cubic Spline Interpolation for Short-Term Gaps

When the time interval of missing values is less than 6 h, the missing integer data are filled by piecewise cubic spline interpolation. Cubic spline interpolation is a commonly used numerical fitting method, which aims to connect the data points continuously with cubic polynomial segments to ensure the continuity of the overall function in the first and second-order derivatives. Let the set of known interpolation points be $\left(x_{0},y_{0}),(x_{1},y_{10}),\ldots ,(x_{n},y_{n}\right)$. A cubic polynomial is constructed for each subinterval $\left[x_{i},x_{i+1}\right]$ as follows:

(7) $$C_{i}\left(x\right)=a_{i}+b_{i}\left(x-x_{i}\right)+c_{i}{\left(x-x_{i}\right)}^{2}+d_{i}{\left(x-x_{i}\right)}^{3},\;\;x\in [x_{i},x_{i+1}]$$

The coefficients $(a_{i}$, $b_{i}$, $c_{i}$, $d_{i}$) are uniquely determined by the following continuity and boundary conditions:

(1) Interpolation condition: $C_{i}\left(x_{i}\right)=y_{i}$, $C_{i}\left(x_{i+1}\right)=y_{i+1}$;

(2) First derivative continuity: ${C_{i}}^{\prime }\left(x_{i+1}\right)={C_{i+1}}^{\prime }\left(x_{i+1}\right)$;

(3) Second derivative continuity: ${C_{i}}^{\prime \prime }\left(x_{i+1}\right)={C_{i+1}}^{\prime \prime }\left(x_{i+1}\right);$

(4) Boundary conditions, e.g., natural spline: ${C_{0}}^{\prime \prime }\left(x_{0}\right)=0,\;{C_{n-1}}^{\prime \prime }\left(x_{n}\right)=0$.

To adapt to the actual acquisition characteristics and interpolation accuracy requirements of buoy trajectory data, this paper introduces the following constraints and supplementary mechanisms in the interpolation strategy. (1) Retain the original hourly data: for the hourly time that already exists in the original record (such as 00:00, 01:00, etc.), directly retain the original values without interpolation to ensure that the data accurately reflects the original state. (2) Approximate hourly processing: considering that some record timestamps may have slight deviations, if the timestamp is within ±1 min of the hourly time (such as 00:00:59, 01:00:01), it is regarded as hourly data to avoid unnecessary interpolation disturbance. (3) Only interpolate missing hourly points: when constructing an equally spaced time series (one per hour), only the missing target hourly points in the original data are interpolated, maintaining the necessity and minimization principle of the interpolation process. (4) Interpolation range restriction: in order to avoid unreliable estimation of time periods far from the actual data points, the maximum extrapolation range is set to 24 h. No interpolation is performed beyond this range, thereby controlling the accumulation of interpolation errors.

#### 3.5.2. Periodicity-Guided Interpolation for Long-Term Gaps

When the time interval between trajectory points exceeds 6 h, the missing segment is treated as long-term data absence, for which simple interpolation may yield unphysical results. To address this, we propose a method that combines dominant periodicity estimation with physical constraints to guide interpolation. Hydrological station measurements indicate that runoff variation was minimal during the study period (January–April 2021), supporting the reasonableness of using the dominant tidal period for interpolation in this river reach. Using hourly-sampled position data, we apply the Welch method for power spectral density (PSD) [33] estimation on the cleaned time series. This method segments the signal, applies fast Fourier transform (FFT) [34,35] to each segment, and averages the resulting periodograms, providing lower variance and better frequency resolution than standard FFT.

The PSD estimate is given as:

(8) $${PSD}_{welch}(f)=\frac{1}{K}\sum_{k=1}^{K}{\left|FFT(x_{k}(t)\cdot w(t))\right|}^{2}$$

where $x_{k}(t)$ is the k-th segment, w(t) is the window function, and K is the number of segments. The dominant frequency $f_{max}$ is extracted from the peak of the PSD, and the corresponding dominant period $T_{main}$ is given as:

(9) $$T_{main}=\frac{1}{f_{max}}$$

In scenarios where the trajectory data are sparse or recorded at irregular timestamps, the Lomb–Scargle periodogram [36] is adopted for dominant frequency identification. This method is particularly suitable for unevenly sampled time series, as it evaluates the goodness-of-fit of sinusoidal models at each candidate frequency without requiring uniform sampling. The Lomb–Scargle power at frequency f is computed as:

(10) $$P(f)=\frac{1}{2\sigma^{2}}[\frac{{\left[\sum_{i}x_{i}cos(2\pi f(t_{i}-\tau ))\right]}^{2}}{\sum_{i}x_{i}{cos}^{2}(2\pi f(t_{i}-\tau ))}+\frac{{\left[\sum_{i}x_{i}sin(2\pi f(t_{i}-\tau ))\right]}^{2}}{\sum_{i}x_{i}{sin}^{2}(2\pi f(t_{i}-\tau ))}]$$

where τ is the phase offset ensuring orthogonality of the basic functions, and $\sigma^{2}$ is the variance of the time series data $\left\{x_{i}\right\}$. The frequency corresponding to the maximum spectral power, denoted $f_{LS}^{max}$, is selected as the dominant frequency when statistically significant. However, if the periodogram does not exhibit a statistically significant peak, the model defaults to the M2 principal lunar semidiurnal tide, which has a period of approximately $T_{M2}=12.42\;h$. Once the dominant period *T* is determined, the timestamp *t* of each data point is mapped to a phase angle on the unit circle to capture the periodic characteristics of motion:


(11) $$\theta (t)=2\pi \cdot \frac{tmodT}{T}$$

The resulting periodic feature vector is defined as:

(12) $$X_{t}^{period}=[sin(\theta (t)),cos(\theta (t))]$$

This 2-dimensional representation ensures rotational invariance and preserves temporal periodicity in a continuous manner. In addition, to account for long-term linear trends in movement (such as net displacement over time), a temporal drift feature is defined as:

(13) $$X_{t}^{trend}=[\frac{t-t_{0}}{∆T}]$$

where $t_{0}$ is the initial timestamp, and ∆T is the total observation window length. The full input vector for regression is thus defined as:

(14) $$X_{t}=[X_{t}^{period},X_{t}^{trend}]$$

A linear regression model can be then constructed:

(15) $${\hat{y}}_{t}=X_{t}\cdot \beta +\varepsilon$$

where ${\hat{y}}_{t}$ is the predicted 2D position at time t, β is the coefficient matrix, and $\varepsilon \sim N(0,\sigma^{2})$ is the residual error. The model parameters are estimated via ordinary least squares (OLS) using historical trajectory data. For missing points, the corresponding timestamp is converted into a periodic feature vector $X_{t}$ and fed into the trained model to obtain predicted positions, achieving interpolation based on learned temporal regularities.

To avoid unrealistic extrapolation during interpolation, especially for moored buoys whose movement is physically bounded by the length of the mooring chain (denoted as L), a distance-based constraint is employed. Let ($x_{ref},y_{ref}$) denote the last known valid position prior to interpolation. The predicted position $(x_{t},y_{t}$) is validated against the following constraint:

(16) $$D_{t}=\sqrt{{\left(x_{t}-x_{ref}\right)}^{2}+{\left(y_{t}-y_{ref}\right)}^{2}}$$

If the displacement exceeds twice the mooring chain length, the interpolated position is considered physically implausible and labeled as a signal loss state:


(17) $$D_{t}>L$$

**Figure 5:**
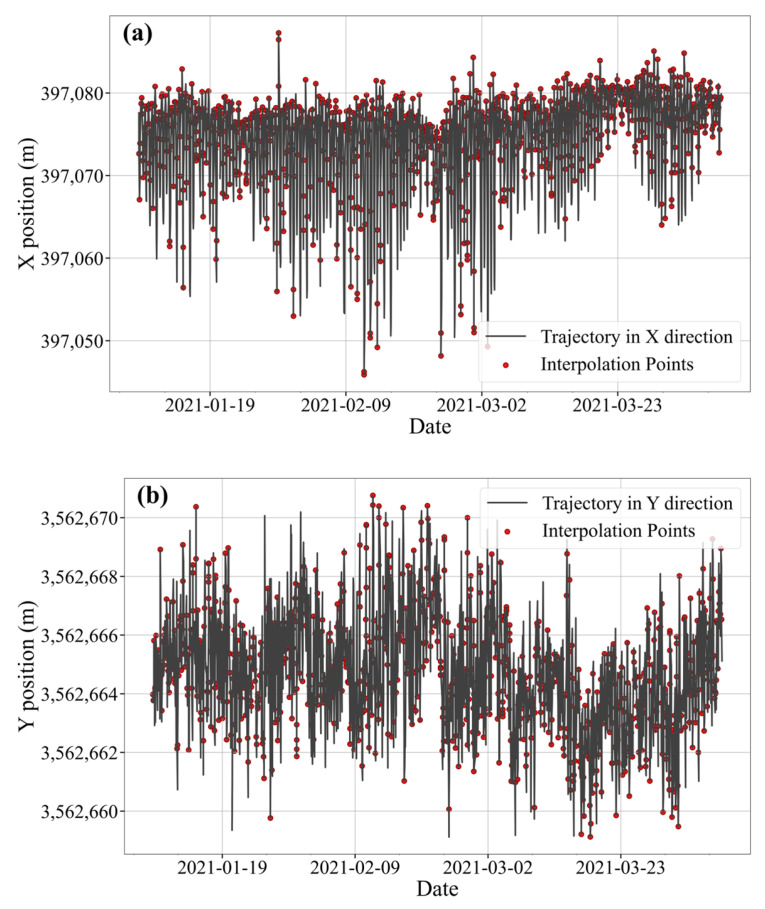

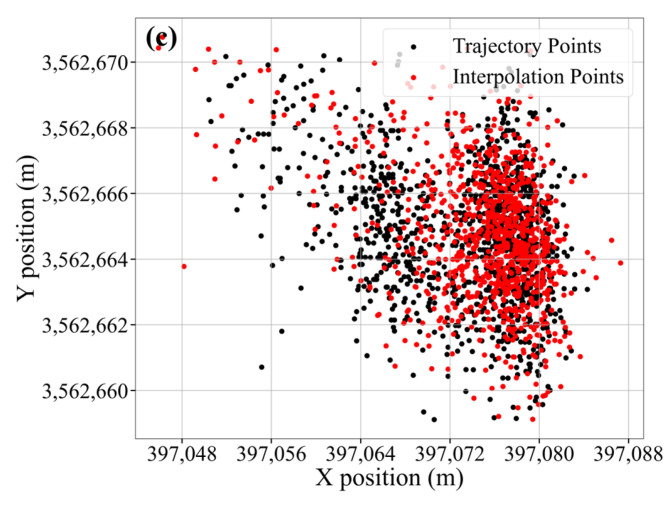
Interpolation points of #135 black buoy. (**a**) Interpolation effect in the X direction (**b**) Interpolation effect in the Y direction (**c**) Interpolation point distribution.

An illustrative example using historical trajectory data of buoy #135 in region S1 is shown in Figure 5, demonstrating the efficacy of the interpolation approach.

### 3.6. Buoy Trajectory Denoising

To reconstruct physically meaningful buoy trajectories from noisy position measurements, we develop an adaptive unscented Kalman filter (AUKF) [14,22] specifically tailored for moored buoy dynamics in an environment with complex tidal runoff hydrodynamic conditions. This method accounts for three key challenges in the lower Yangtze River: (1) periodic tidal-induced motion, (2) abrupt shifts caused by flow transitions or mechanical disturbances, and (3) varying signal noise over time. These issues are addressed by embedding mode-aware dynamics, adaptive noise modeling, and innovation-driven state correction into the filtering framework. In particular, the AUKF introduces a covariance adaptation mechanism based on innovation statistics within the nonlinear UKF framework. The algorithm can automatically balance the weight ratio between state prediction and observation updates by adjusting the covariance of the state and observation vectors in real-time, thereby improving system robustness [37] and filtering accuracy under non-Gaussian or time-varying noise conditions. Furthermore, we incorporate the Singer acceleration model into the state-space representation, which effectively captures the correlated acceleration behavior of realistic buoy motion. This integration enhances the model’s ability to represent trajectory dynamics with higher stability and physical fidelity.

#### 3.6.1. State Vector Design and Physics-Informed Dynamic Modeling

The buoy motion on a horizontal plane is represented by a six-dimensional continuous state vector:

(18) $$X(t)={\left[x(t),v_{x}(t),a_{x}(t),y(t),v_{y}(t),a_{y}(t)\right]}^{T}$$

where $\left(x(t),y(t)\right)$ is the spatial position in projected coordinates; $\left(v_{x}(t),v_{y}(t)\right)$ represents the instantaneous velocities; and $\left(a_{x}(t),a_{y}(t)\right)$ denotes accelerations, representing the resultant force from tides, currents, and wind disturbances.

This model is designed to simultaneously capture long-term trends (e.g., tidal drifts) and short-term perturbations (e.g., wave-induced jumps). Compared to models only considering position or velocity, the inclusion of acceleration states provides a better representation of system inertia and external force response. The Singer acceleration model is employed, where acceleration evolves as a first-order Markov process with exponential decay:

(19) $$a(t+∆t)=a(t)\cdot e^{-\alpha ∆t}$$

Here, α is a maneuverability parameter determining the decay rate. Smaller values of α correspond to smooth, inertia-dominant motion (tidal drift), while larger *α* values model rapid, high-response dynamics (abrupt perturbations). The Singer model provides a more realistic representation of correlated acceleration behavior than traditional constant-velocity (CV) or constant-acceleration (CA) Kalman filters, while retaining a relatively simple linear Gaussian structure. It effectively captures short-term maneuvering and oscillatory motion, which are common in moored buoy trajectories influenced by tides and flow transitions. The position and velocity of the x-axis transition over a time step ∆t is:

Position update:

(20) $$x(t+∆t)=x(t)+v_{x}(t)\cdot ∆t+a_{x}(t)\cdot \frac{\alpha ∆t-1+e^{-\alpha ∆t}}{\alpha^{2}}$$

Velocity update:

(21) $$v_{x}(t+∆t)=v_{x}(t)+a_{x}(t)\cdot \frac{1-e^{-\alpha ∆t}}{\alpha }$$

The same formulation applies to the y-axis by replacing variables accordingly.

#### 3.6.2. Multi-Mode Dynamic Modeling and Switching Mechanism

Given that hydrodynamic forces acting on buoys vary across tidal cycles and local disturbances, a mode-aware modeling structure is incorporated that dynamically adapts to different motion regimes. The filter autonomously switches between predefined dynamic modes based on innovation statistics, enhancing estimation accuracy under non-stationary conditions. The motion modes are defined as follows (Table 2):

Each mode is characterized by the decay rate to control the dynamic responsiveness, and the process noise variance to capture unmodeled accelerations.

#### 3.6.3. Noise Modeling and Innovation-Based Mode Adaptation

The state evolution [22] includes process noise w(t), primarily accounting for unpredictable environmental disturbances. The discrete-time process noise covariance $Q\in R^{6\times 6}$ is constructed from:

(22) $$G=\left[\begin{array}{c} \frac{\alpha ∆t-1+e^{-\alpha ∆t}}{\alpha^{2}}\\ \frac{1-e^{-\alpha ∆t}}{\alpha }\\ 1 \end{array}\right]$$

(23) $$Q=\sigma_{a}^{2}\cdot G\cdot G^{T}$$

where $\sigma_{a}^{2}$ is the standard deviation of acceleration noise, and its values in different modes are set to tidal mode $\sigma_{a}=0.03\;m/h^{2}$, mixed mode $\sigma_{a}=0.1\;m/h^{2}$ and abrupt mode $\sigma_{a}=0.5\;m/h^{2}$. Only 2D positions are observed, and measurement noise varies due to buoy orientation, environmental factors, and receiver conditions. We use a diagonal measurement noise covariance R with variance values that increase under abrupt modes, tolerating possible sensor outliers or multipath effects.

**Figure 6:**
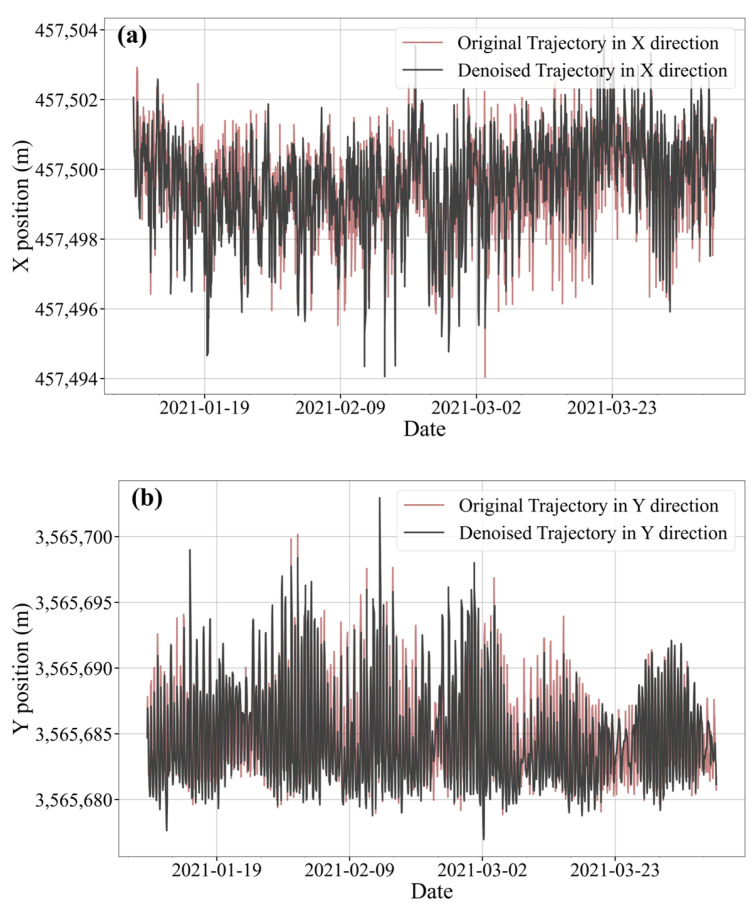

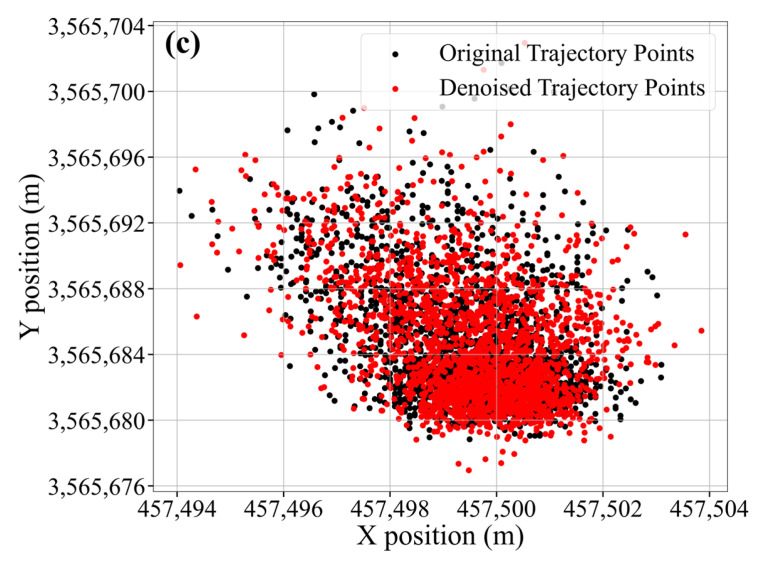
Comparison of #104 red buoy data before and after denoising. (**a**) Noise reduction effect in the X direction (**b**) Noise reduction effect in the Y direction (**c**) Distribution of noise reduction points.

At each step, the innovation vector (observation residual) is:

(24) $${\tilde{y}}_{t}=z_{t}-{\hat{z}}_{t}$$

with normalized innovation magnitude computed as:

(25) $$\gamma_{t}={{\tilde{y}}_{t}}^{T}S^{-1}{\tilde{y}}_{t}$$

where *S* is the innovation covariance matrix. A sliding window average ${\bar{\gamma }}_{t}$ over the last 5 steps is maintained, and switch modes based on different thresholds. If ${\bar{\gamma }}_{t}<0.25$, switch to tidal mode, switch to abrupt mode if ${\bar{\gamma }}_{t}>1.3$. Otherwise, it will remain in mixed mode. Small values indicate motion dominated by regular tides, while large values reflect abrupt disturbances. The thresholds were empirically tuned and validated on 295 buoys across 9 river sections to ensure responsiveness and robustness. Taking the #104 red buoy in S3 as an example, after the AUKF is processed, the comparison chart is shown in Figure 6.

As can be seen from the figure, the red original data shows a lot of high-frequency noise and spikes, while the black filtered data is obviously smoother, because the extreme values and mutation points in the original data are filtered and noise-reduced. The original data in the X direction has a high noise level, and the AUKF processing maintains the main trend while significantly reducing the noise amplitude; compared with the X direction, the original noise in the Y direction seems to be slightly smaller, and the filtering effect is also significant, the smoothness is greatly improved, but at the same time the main characteristics and trends of the original data are well maintained.

### 3.7. Feature Extraction

#### 3.7.1. Time-Domain Features

To characterize the fundamental spatial properties of buoy trajectories, a series of descriptive statistical measures, including centrality, directional dispersion, and higher-order moment statistics are computed. The initial deployment location is defined as $\left(x_{0},y_{0}\right)$, based on the trajectory data comprising n timestamped positions $\left(x_{i},y_{i}\right)$ The empirical centroid of the buoy’s trajectory is calculated as:

(26) $$\bar{x}=\frac{1}{n}\sum_{i=1}^{n}x_{i},\bar{y}=\frac{1}{n}\sum_{i=1}^{n}y_{i}$$

The radial distance set ${\left\{r_{i}\right\}}_{i=1}^{n}$ can be then calculated by:

(27) $${r_{i}=\sqrt{{\left(x_{i}-\bar{x}\right)}^{2}+{\left(y_{i}-\bar{y}\right)}^{2}}}_{i=1}^{n}$$

Different radial distribution parameters, such as maximum radial drift distance, mean radial drift distance and standard deviation of radial distances can be derived as [38]:

(28) $$r_{max}=max(r_{i})$$

(29) $$\bar{r}=\frac{1}{n}\sum_{i=1}^{n}r_{i}$$

(30) $$\sigma_{r}=\sqrt{\frac{1}{n}\sum_{i=1}^{n}{\left(r_{i}-\bar{r}\right)}^{2}}$$

These metrics describe the spatial dispersion of the buoy around its empirical center of movement. To assess directional variability, the standard deviation along the X and Y axes can be described as:

(31) $$\sigma_{x}=\sqrt{\frac{1}{n}\sum_{i=1}^{n}{\left(x_{i}-\bar{x}\right)}^{2}}$$

(32) $$\sigma_{y}=\sqrt{\frac{1}{n}\sum_{i=1}^{n}{\left(y_{i}-\bar{y}\right)}^{2}}$$

The axis ratio $R_{xy}=\frac{\sigma_{x}}{\sigma_{y}}$ quantifies the anisotropy of movement. A value $R_{xy}>1$ indicates stronger motion along the X-axis, whereas $R_{xy}<1$ suggests a dominant Y-axis trajectory.

To capture non-Gaussian characteristics in the buoy motion distribution, the skewness and kurtosis of the coordinate time series are calculated.

(33) $$S_{x}=\frac{\frac{1}{n}\sum_{i=1}^{n}{\left(x_{i}-\bar{x}\right)}^{3}}{{\sigma_{x}}^{3}}$$

(34) $$S_{y}=\frac{\frac{1}{n}\sum_{i=1}^{n}{\left(y_{i}-\bar{y}\right)}^{3}}{{\sigma_{y}}^{3}}$$

(35) $$K_{x}=\frac{\frac{1}{n}\sum_{i=1}^{n}{\left(x_{i}-\bar{x}\right)}^{4}}{{\sigma_{x}}^{4}}-3$$

(36) $$K_{y}=\frac{\frac{1}{n}\sum_{i=1}^{n}{\left(y_{i}-\bar{y}\right)}^{4}}{{\sigma_{y}}^{4}}-3$$

These higher-order moments help identify asymmetric drift (via skewness) and peaked or flattened trajectory distributions (via kurtosis), which can catch signal aggregation tendencies and irregular movements.

To further analyze the spatial pattern of buoy motion, density-based clustering using the DBSCAN (density-based spatial clustering of applications with noise) algorithm is employed. This method partitions the trajectory into high-density regions without requiring predefined cluster counts, using two key parameters, neighborhood radius ε and the minimum number of points MinPts. After clustering, the silhouette coefficient is used to assess clustering quality:

(37) $$s=\frac{1}{n}\sum_{i=1}^{n}\frac{b_{i}-a_{i}}{max(a_{i},b_{i})}$$

Here, $a_{i}$ is the average intra-cluster distance, and $b_{i}$ is the minimum average distance to points in neighboring clusters. A higher silhouette score indicates better-defined and more compact clusters. The centroid of each identified cluster is then calculated and weighted by local point density to obtain the density-weighted cluster center $\left(x_{c},y_{c}\right)$. The maximum and mean distance to the cluster center can be expressed as:

(38) $$r_{max,cluster}=max{\left\{\sqrt{{\left(x_{i}-x_{c}\right)}^{2}+{\left(y_{i}-y_{c}\right)}^{2}}\right\}}_{i\in C}$$

(39) $${\bar{r}}_{cluster}=\frac{1}{\left|C\right|}\sum_{i\in C}\sqrt{{\left(x_{i}-x_{c}\right)}^{2}+{\left(y_{i}-y_{c}\right)}^{2}}$$

These parameters reflect the internal spread of each motion cluster and provide insight into the spatial density and consistency of buoy trajectories.

Furthermore, to quantify the overall mobility and identify outlier displacements, the cumulative distribution function (CDF) of radial distances $r_{i}$ is analyzed. And use F(r)=P(R≤r) to denote the empirical CDF, from which a multi-level quantile set is derived:

(40) $$Q=\{q_{0.00},q_{0.25},q_{0.50},q_{0.75},,q_{0.85},q_{0.90},q_{0.95},q_{0.98},q_{0.99},q_{1.00}\}$$

These quantiles characterize the statistical range and extremity of buoy displacements. Specifically, upper quantiles such as $q_{0.95}$ and above are useful for detecting sudden drift anomalies, while interquartile ranges help define typical operational movement zones.

#### 3.7.2. Time-Frequency Features Extraction Based on Wavelet Transform

It is difficult to capture non-stationarity, local anomalies, and frequency changes by relying solely on time-domain statistical features. Therefore, it is necessary to decompose multiple scales by combining the wavelet transform [39,40], detecting the mutation points of the buoy, and conducting joint analysis in the time domain and frequency domain [41].

To capture the non-stationary and multiscale motion behavior of river buoys induced by multiple physical drivers (e.g., abrupt collisions, tidal oscillations, and background runoff), this study constructs a wavelet-based feature system using a discrete wavelet transform (DWT). Wavelets provide adaptive time-frequency resolution, making them suitable for signals with both abrupt events and periodic oscillations, whereas alternatives like STFT or EMD face limitations in low-frequency resolution or noise sensitivity. The buoy displacement time series x(t) and y(t) are decomposed into multiple levels of detail coefficients $d_{j}(t)$, each corresponding to specific temporal scales. The decomposition levels are mapped to hydrodynamically relevant scales as follows:

1. Small scales (levels 1–3) represent fast fluctuations over minutes to a few hours, reflecting transient events such as vessel passage, physical collisions, or sensor disturbances.

2. Medium scales (levels 5–7) correspond to semidiurnal (12.42 h) and diurnal (24.84 h) tidal cycles, capturing periodic tidal motions.

3. Large scales (levels ≥ 9) capture low-frequency components over multi-day periods (e.g., >72 h), associated with background runoff, wind-driven currents, or broader circulation patterns.

For each scale j, the energy of the corresponding detail coefficients is calculated as:

(41) $$E_{j}=\sum_{t}{\left|d_{j}(t)\right|}^{2}$$

which represents the total power contribution of that frequency band.

To quantify the strength and frequency of transient motion events, we compute the local energy variation rate $∆E_{j}(t)$ at small scales (levels 1–3) and define the maximum rate as the abrupt change intensity:

(42) $$v_{abrupt\_change}={max}_{t}\left|\frac{d}{dt}E_{j}\left(t\right)\right|,j\epsilon \{1,2,3\}$$

To detect discrete events, a dynamic threshold is defined based on statistical deviation:

(43) θ=μ+2σ

where μ and σ are the mean and standard deviation of the local energy variation rate. Points where $∆E_{j}(t)>\theta$ are flagged as abrupt events, and the total count is recorded as:

(44) $$v_{event\_count}=sum\{t|∆E_{j}(t)>\theta \}$$

To characterize the dominant physical drivers behind buoy motion, we calculate the relative energy ratio at medium and large scales:

(45) $$v_{j}=\frac{E_{j}}{\sum_{k}E_{k}}$$

These ratios reflect how strongly the buoy motion is affected by periodic tidal processes versus slowly varying background flow. Finally, the dominant energy scale is identified by the level j with the highest energy:

(46) $$v_{dominant\;oscillation}=arg{max}_{j}E_{j}$$

This compact descriptor reveals the most influential frequency band driving the buoy’s overall dynamics, aiding pattern recognition and classification. The features extracted are as follows (Table 3).

**Figure 7:**
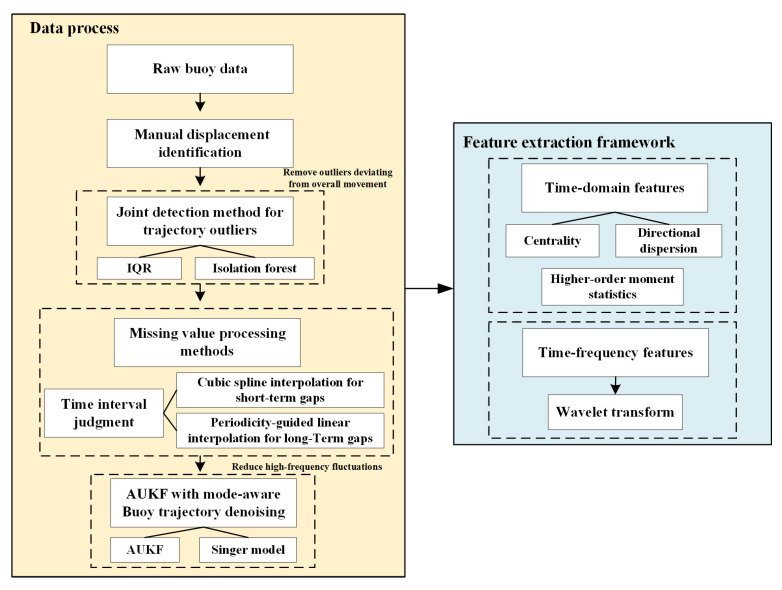
Methodology of data processing and feature extraction.

The methodology of data processing and feature extraction can be simply shown as a schematic diagram (Figure 7).

The proposed framework has been effectively applied to buoy data from the lower Yangtze River, producing reliable trajectory datasets and revealing essential motion patterns. It can provide practical support for intelligent buoy monitoring and enhance inland waterway safety and management efficiency.

## 4. Analysis of Buoy Motion Characteristics

### 4.1. Representative Buoy Selection via Trajectory Similarity

To ensure that the selected buoys can accurately reflect the characteristic motion patterns of each navigational segment, a trajectory similarity analysis method based on dynamic time warping (DTW) is employed. DTW [42] is a widely-used time series alignment technique capable of measuring similarity between sequences that may vary in speed or temporal scale. Given two time series sequences $A=\{a_{1},a_{2},\ldots ,a_{n}\}$ and $B=\{b_{1},b_{2},\ldots ,b_{n}\}$, DTW constructs a warping path $W=\{(i_{1},j_{1}),(i_{2},j_{2}),\ldots ,(i_{K},j_{K})\}$, where $i_{K}\in \left[1,n\right],\;j_{K}\in [1,n]$, and K is the length of the path. The goal is to find the optimal alignment that minimizes the cumulative distance:

(47) $$DTW\left(A,B\right)={min}_{w}\{\sum_{k=1}^{K}d(a_{ik},b_{jk})\}$$

where $d(a_{ik},b_{jk})$ is the local distance, often defined as the squared Euclidean distance $d\left(a_{i},b_{j}\right)={\left(a_{i}-b_{j}\right)}^{2}$. The warping path W is subject to constraints ensuring boundary conditions (W starts and ends at corresponding ends of A and B), monotonicity (time ordering is preserved), and continuity (no time steps are skipped).

For each navigational segment {S1~S9}, pairwise DTW distances between the trajectories of all buoys located within that segment can be obtained. Since buoy trajectories are two-dimensional (x and y coordinates), the DTW distances are calculated separately in each direction. Let ${DTW}_{x}^{\left(i,j\right)}$ and ${DTW}_{y}^{\left(i,j\right)}$ denote the DTW distances between buoy i and buoy j in the x and y directions, respectively. For each buoy i, the average DTW distance to all other buoys in the same segment can be computed as:

(48) $${DTW}_{x}^{\left(i\right)}=\frac{1}{N-1}\sum_{j=1,j\neq i}^{N}{DTW}_{x}^{\left(i,j\right)}$$

(49) $${DTW}_{y}^{\left(i\right)}=\frac{1}{N-1}\sum_{j=1,j\neq i}^{N}{DTW}_{y}^{\left(i,j\right)}$$

where N is the total number of buoys in the segment. Then, a composite similarity indicator for each buoy as the Euclidean combination of the directional similarities can be defined as:

(50) $${Dist}^{\left(i\right)}=\sqrt{({DTW}_{x}^{\left(i\right)}{)}^{2}+({DTW}_{y}^{\left(i\right)}{)}^{2}}$$

This indicator reflects the average deviation of buoy i’s trajectory from others in the same segment, accounting for both spatial dimensions. Therefore, the buoy with the minimum composite DTW distance is selected as the representative buoy, as it demonstrates the highest overall similarity to all others in that segment.

### 4.2. Results and Discussion

#### 4.2.1. Extracted Features Analysis of Multiple Segments

Following the DTW-based representative buoy selection, a comprehensive analysis of the motion characteristics of each selected buoy is conducted across nine navigational segments. Red buoys, typically cylindrical or downward-pointing conical in shape, indicate the left side of the channel (when proceeding upstream), while black buoys, usually conical, indicate the right side. The number preceding the color indicates its identification number, and the numbers in the lower reaches of the Yangtze River decrease in sequence. The letters before the numbers indicate that the buoy has special functions, locations, etc. These features provide insights into the hydrodynamic consistency and spatial regularity of different segments. The results are summarized in Table 4.

The average spatial coordinates $\bar{x}$ and $\bar{y}$ show that buoys are well distributed across the longitudinal and latitudinal range of the river, the error between the coordinate centroid and the preset location is small. The centroid positions $\left(x_{c},y_{c}\right)$ computed from the trajectories are highly consistent with $\left(\bar{x},\bar{y}\right)$, confirming the representativeness of the buoy within each segment.

The values of $r_{max}$ and $\bar{r}$ quantify the maximum and average displacement of buoys from their centroid. Segment $S_{9}$ exhibits the largest movement range ($r_{max}=56.25\;m,\bar{r}=18.60\;m$), whereas $S_{3}$ and $S_{1}$ present more limited motion ($r_{max}<18\;m,\bar{r}<3.5\;m$). This suggests that the buoy motion amplitude varies significantly across segments. The buoy displacement in the downstream section (S7~S9) is significantly larger, which may be due to the stronger current or more complex hydrodynamics in the estuary, resulting in an expansion of the buoy activity range. The average displacement $\bar{r}$ and the standard deviation $\sigma_{r}$ generally increase downstream. This shows that the downstream buoys not only have a larger overall displacement but also experience more dramatic fluctuations in all directions, indicating that the flow field is more complex and the forces change frequently.

The axis ratio $R_{xy}$ reveals the dominant drift direction of buoys, reflecting the local flow field geometry. The upstream buoys exhibit more lateral deviations, while the downstream buoys move along the main heading (longitudinal direction), dominated by tides or current speed. For instance, in S1, S2, S5 and S7, $R_{xy}>1$, the dominant direction of hydrodynamics is the X direction. While the remaining sections are dominated by the Y direction, suggesting significant longitudinal tidal components or vertical turbulent disturbances in these areas.

Skewness and kurtosis reveal the asymmetry and peak behavior of movement. In terms of skewness, the mid- and downstream sections S3, S4, S7–S9 all showed rightward deviation in the Y direction, indicating that these areas were more prone to tail deviation in the downward direction. S4 had the largest deviation (1.65), which may be related to strong tidal currents/longitudinal impacts. The upstream S1 showed leftward deviation, with the movement more concentrated upstream. For kurtosis, S1 has high peaks (>4) in both X and Y directions, indicating that its movement is extremely concentrated and has small fluctuations. Downstream segments (such as S8 and S9) show flatter peak characteristics, indicating that their trajectory drifts over a wide range and is relatively evenly distributed. It can be inferred that in the controlled flow environment, the upstream buoys, such as S1–S2, have concentrated motion and small excursion ranges, while the downstream segments, such as S7–S9, are more unstable due to external forces and have more dispersed trajectories.

After multi-track clustering, the downstream buoys still show a larger range of motion, indicating that this difference was systematic rather than accidental in a single track. Not only does the movement trajectory expand overall, but the distribution range also increases systematically at the median and extreme values, and there are more frequent and larger displacement events downstream. The metrics provide a robust statistical view of the drift envelope. For instance, S9 has the widest 99th-percentile range ($q_{0.98}=45.85\;m$), while S3 and S1 maintain narrower distributions.

#### 4.2.2. Extracted Features Analysis of Single Segments

**Figure 8:**
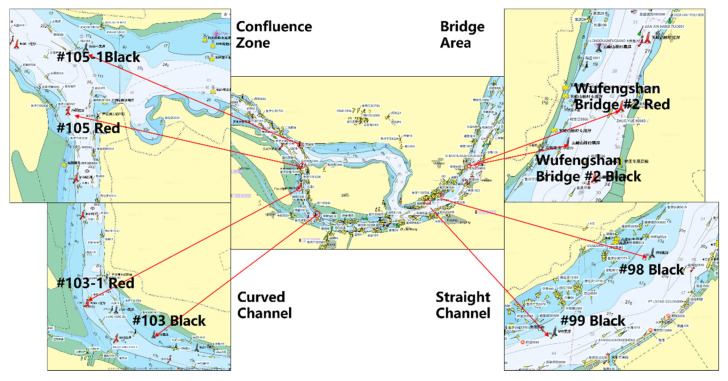
Representative buoy’s location.

Even within the same waterway, buoys may exhibit different motion patterns due to variations in their surrounding geographical environment and navigational conditions. To examine such localized differences in greater detail, this section focuses on S3. Representative buoys located at different waterway environments—including curved channels, straight channels, confluences, and bridge areas—were selected for analysis (their spatial distribution is shown in Figure 8), and the corresponding motion parameters are summarized in Table 5.

Based on the analysis of motion parameters for representative buoys within S3, significant differences in buoy motion characteristics are observed under varying hydrodynamic and environmental conditions. Statistical comparisons were conducted among buoys located at straight channels (#99black, #98black), curved channels (#103-1red, #103black), confluence zones (#105-1black, #105red), and bridge areas (Wufengshan Bridge #2red, #2black), revealing a clear correlation between buoy stability and surrounding waterway morphology.

In terms of displacement range, buoys deployed in straight and curved channels exhibit relatively high motion stability, with maximum displacements ranging from 14.40 m to 32.20 m and average displacements between 3.48 m and 5.50 m. Among them, the #103-1red buoy demonstrates the highest stability, with the minimum observed maximum displacement of 14.40 m. Buoys in the confluence zone, affected by flow bifurcation, show comparatively larger motion ranges, with a maximum displacement of 25.98 m and an average displacement of 5.30 m. Bridge-area buoys experience the most complex forces due to the combined effects of flow obstruction from bridge piers and vessel-induced disturbances. These buoys show maximum and average displacements of 40.82 m and 5.85 m, respectively.

Analysis of distributional characteristics further reveals directional differences in buoy motion induced by varying waterway types. Buoys in straight channels display wider variation in the XY ratio (1.07–1.68), suggesting more prominent motion along the X direction. Curved-channel buoys exhibit relatively stable XY ratios (0.96–1.65), comparable in magnitude to those in straight channels, indicating relatively concentrated motion. In contrast, buoys in the confluence and bridge zones present significantly larger standard deviations in the Y direction and higher kurtosis values, indicating stronger and more directional perturbations in their trajectories. This pattern is further supported by quantile-based analysis. Buoys in straight and curved channels exhibit generally lower 95th percentile displacement values (Q95 ranging from 7.60 m to 15.96 m), while buoys in the confluence and bridge zones show higher Q95 values (12.19 m to 17.23 m). This suggests that buoys in complex environments are more likely to experience frequent and large-magnitude displacements, reflecting reduced positional stability under compounded hydraulic influences.

#### 4.2.3. Analysis of Features Based on Wavelet Transform

The wavelet transform characteristics of the representative buoys of each segment are calculated, and the results are presented in the following Table 6.

**Figure 9:**
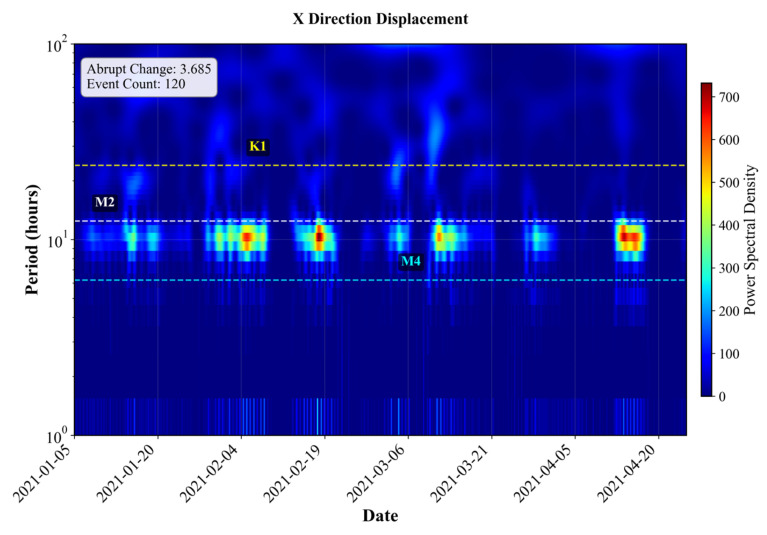

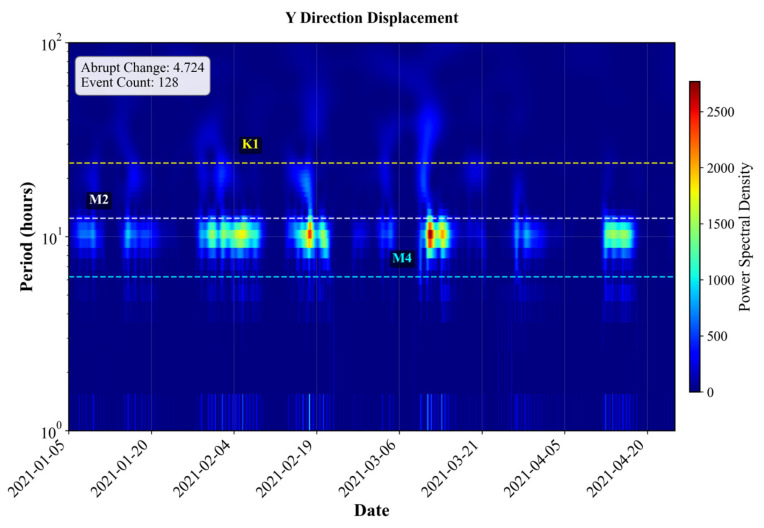
Wavelet transform result of #80 red buoy (S4).

**Figure 10:**
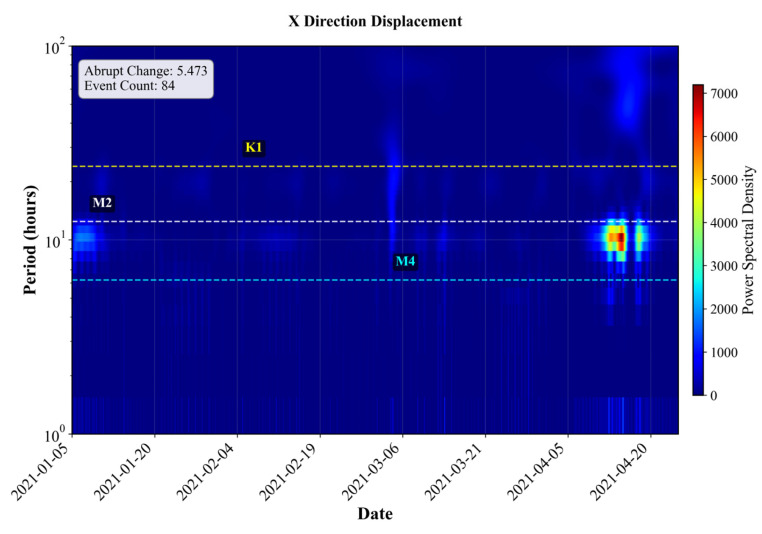

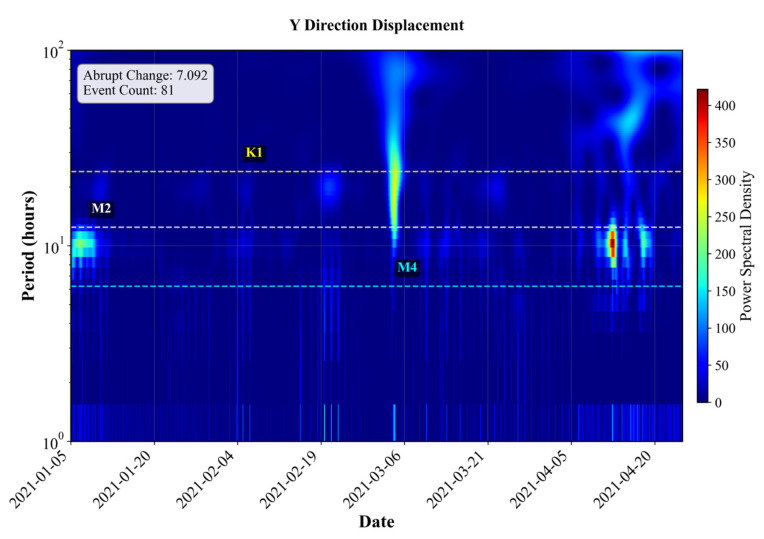
Wavelet transform result of #68 black buoy (S5).

Based on wavelet-derived features from nine representative segments, a clear directional asymmetry is observed in abrupt motion patterns. The abrupt change intensity in the Y direction is generally higher than that in the X direction, ranging from 1.74 to 7.09, compared with ranges from 2.75 to 5.47 in the X direction. This indicates that vertical (Y-axis) buoy motion is more susceptible to sudden hydrodynamic disturbances. The energy ratio analysis reveals a strong dominance of tidal dynamics across all segments. The semidiurnal tidal energy (M2) accounts for the majority of signal energy in both directions, with X-direction values ranging from 0.93 to 6.19, and Y-direction values spanning from 0.55 to 9.65, reflecting higher vertical variability. In contrast, diurnal (K1/O1) and runoff-related energy components are relatively minor, typically within 0.4–1.4 and 0.6–1.6, respectively. The tidal-to-runoff energy ratio further confirms the dominance of tidal forcing, with most segments exhibiting ratios above 1, signifying that tidal energy exceeds that of background runoff. Furthermore, dominant oscillation mode analysis shows that both X and Y components are primarily governed by semidiurnal tidal oscillations (M2) in most buoys. To illustrate the directional hydrodynamic heterogeneity, two representative buoys in S4 (#80 red buoy) and S5 (#68 black buoy) are selected for detailed comparison based on their wavelet transform results shown in Figure 9 and Figure 10.

Based on wavelet transform analysis, the #80 red buoy located in S4 exhibits a distinct semidiurnal tidal characteristic in both X and Y directions. In the X direction, the signal displays a pronounced tidal modulation with a sharpness index of 3.68 and 120 detected events. The wavelet spectrum shows a clear concentration of energy in the M2 frequency band (~12.42 h), with a localized energy peak reaching approximately 700, indicative of a strong semidiurnal tidal forcing. Additionally, the presence of enhanced energy in the M4 band (~6 h) reveals nonlinear tidal constituents or over tides. The overall energy distribution is spatially localized, suggesting a well-defined and periodic tidal influence. In the Y direction, the sharpness index increases to 4.72 with 128 events, and the wavelet spectrum presents pronounced energy bands in the M2 region, with maxima exceeding 2500.

In contrast, #68 black buoy in S5 displays direction-dependent hydrodynamic behavior. In the X direction, the signal retains a strong semidiurnal tidal pattern, with a sharpness index of 5.47 and 84 detected events. The M2 band shows significant energy concentration with localized high-energy patches exceeding 7000, indicative of a dominant periodic tidal forcing. Minor energy enhancement in the M4 band further supports the presence of harmonic tidal components. The energy is highly localized and periodic, confirming that the X direction is predominantly modulated by tidal dynamics. However, in the Y direction, the sharpness index markedly increases to 7.09 with 81 events, reflecting more abrupt local changes. The spectrum reveals a broad and continuous energy distribution in the low-frequency band (>24 h), consistent with persistent riverine flow forcing. Compared to the X direction, energy in the M2 band is less localized and lacks distinct peaks, implying a weaker or masked tidal signal. The spectrum exhibits a diffuse energy structure characteristic of non-periodic, continuous fluvial processes.

These directional differences highlight the spatial heterogeneity of hydrodynamic conditions, with S4 being predominantly tide-driven in both directions, while S5 demonstrates a clear tidal–fluvial interaction with directional differentiation—tide-dominant in the X direction and river-flow-dominant in the Y direction. This finding is consistent with the study by [43], which reported that semi-diurnal and diurnal tidal constituents propagate in different directions along the coast, and the M2 tide is the dominant tidal constituent. It showed that river discharge significantly dampens tidal amplitudes, particularly in the midstream of the tidal river.

#### 4.2.4. Analysis of Large Displacement Occurrence Time

**Figure 11:**
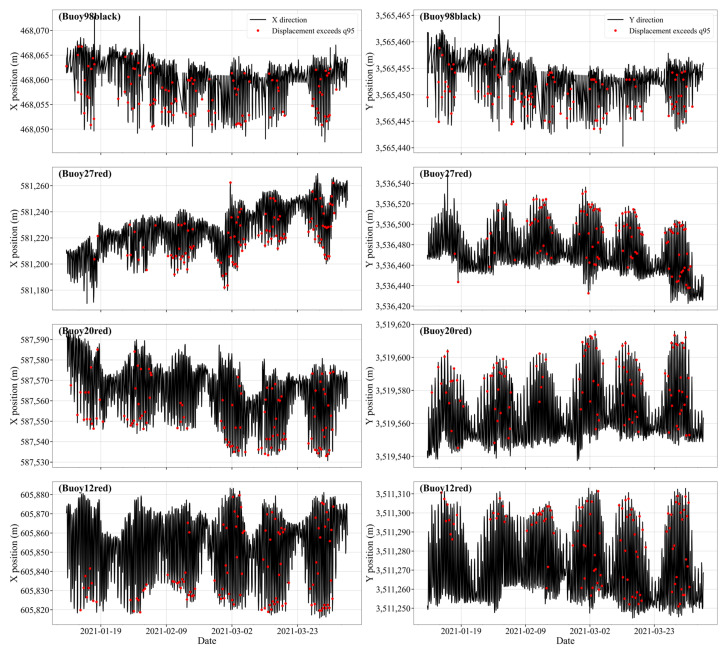
Large displacement occurrence time distribution.

To further verify the spatiotemporal patterns of large buoy displacements, a time series analysis was conducted focusing on abnormal movement events across different channel segments. Specifically, we examined the temporal distribution of displacement events exceeding the 95th percentile threshold of each buoy’s motion distance. Here, the displacement refers to the combined motion distance calculated from both X and Y directions, rather than a single direction. A set of representative buoys was randomly selected from S3 and 7–9 for anomaly analysis, including buoy #98 black buoy (S3), #27 red buoy(S7), #20 red buoy (S8), and #12 red buoy (S9). Their spatial–temporal movements in X or Y directions are illustrated in the Figure 11 below.

**Figure 12:**
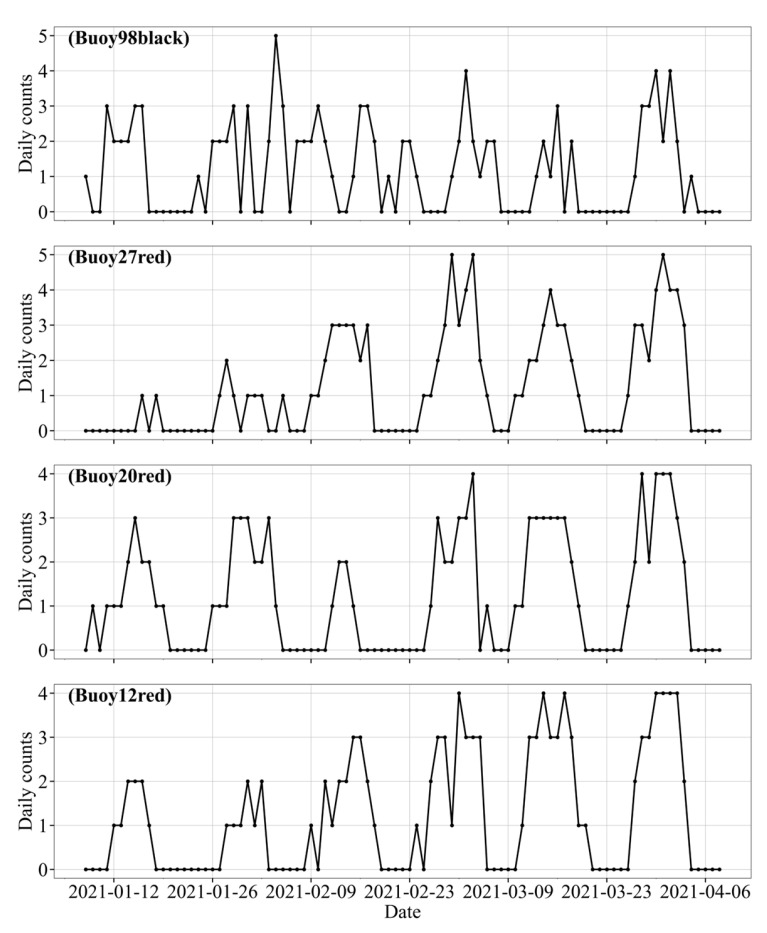
Date distribution of large displacement occurrences.

Despite the differences in geographic location and deployment context, the timing of large displacement events for all four buoys exhibits strong similarities, characterized by distinct periodicity and clustering. To quantitatively validate this observation, the dates of displacement events exceeding the 95th percentile for each buoy were extracted and compared. The detailed temporal distributions are presented in the following Figure 12.

The time distribution shows that large displacement events for all buoys are concentrated and suggest a semi-monthly periodic pattern. These periods coincide closely with the spring-neap tidal cycles of the lunar calendar (new moon and full moon phases). Notably, buoy #20 red and #27 red exhibit the most regular semi-monthly periodic pattern, with peak intervals of approximately 14–15 days, aligning well with the theoretical spring tide cycle of 14.8 days. Although buoy #12 red demonstrates increased displacement activity in the later observation period (March to April), the timing of its peak events remains consistent with that of the other buoys. This strong inter-segment temporal synchronicity suggests that the observed large displacement events are not isolated disturbances caused by local conditions, but rather reflect the influence of broader regional hydrodynamic forcing. Given the alignment between abnormal displacement timings and tidal phases, we infer that tidal forcing is a major factor driving significant buoy motion across multiple waterway segments.

## 5. Conclusions

Buoy motion data in inland waterways often contain high noise levels, outliers, and missing values, complicating their direct application in monitoring and anomaly detection. This study aims to construct a robust data preprocessing and feature extraction framework tailored to buoy trajectory data. To address the challenges of noisy and incomplete time series, we proposed a hybrid preprocessing approach. For outlier detection, a method combining IQR and Isolation Forest is proposed. For missing data imputation, interpolation methods are adaptively selected based on time intervals. For short-term gaps, cubic spline interpolation is applied; otherwise, an interpolation method that combines dominant periodicity estimation with physical constraints based on PSD is proposed. For denoising, an AUKF integrated with the Singer motion model is employed. After data refinement, a set of multiple domain features is extracted; these describe motion, including statistical features, clustering features, and wavelet transform-based features.

The extracted features reveal clear spatial heterogeneity in buoy motion patterns across different channel segments. Statistical time-domain features, such as standard deviation, range, and peak-to-mean ratio, indicate that buoys in downstream segments exhibit larger motion amplitudes and higher volatility, reflecting stronger and more complex hydrodynamic forces near estuarine zones. In general, motion variability increases toward the estuary. Wavelet-based time-frequency analysis further shows that semi-diurnal tides are the dominant driver of buoy motion across most segments, with energy ratios in the M2 band significantly exceeding those of diurnal tides or low-frequency runoff-related components. The Y direction typically exhibits greater mutation intensity and more frequent transient disturbances, suggesting directional sensitivity to external forcing such as wind and tidal shear. Segment-wise comparison of representative buoys highlights directional asymmetry and mixed tidal–runoff interactions, particularly in mid-to-lower reaches. These findings demonstrate the ability of the proposed preprocessing and feature extraction pipeline to capture spatial and temporal variations in buoy dynamics, thereby supporting applications in anomaly detection and intelligent inland waterway monitoring.

While the proposed framework effectively captures key statistical and time-frequency features of buoy motion, it currently lacks a thorough exploration of nonlinear dynamic characteristics. Nonlinear behaviors—such as long-range dependence, chaotic fluctuations, and scaling patterns—are likely to be present in buoy trajectories due to the interplay of multiple natural forces (e.g., tides, currents, and wind). However, these aspects remain underexplored in this study. Future work will incorporate nonlinear descriptors such as fractal dimension, Lyapunov exponents, and entropy-based measures to provide a more comprehensive understanding of buoy dynamics and environmental responses. Such efforts will further reveal the complex motion signatures of buoys and enhance insight into how buoys respond to dynamic inland waterways.

## Figures and Tables

**Table 1:** Major sub-reaches of the lower Yangtze River.

Channel Name	Label
Longtan Channel	S1
Yizheng Channel	S2
Hechangzhou Channel	S3
Kou’an Straight Channel	S4
Taixing–Jiangyin Channel	S5
Fujiasha Channel	S6
Liuhaisha–Nantong Channel	S7
Tongzhousha Channel	S8
Baimosha–Liuhe Channel	S9

**Table 2:** Motion modes definition.

Mode	Label	Physical Interpretation	Decay Rate α (1/h)	Process Noise $\mathrm{Variance}\;\sigma_{a}^{2}$
Tidal	M1	Smooth tidal drift	1/12.4	0.03
Mixed flow	M2	Moderate disturbance period	1/7	0.1
Abrupt	M3	Sudden displacement events	1/2	0.5

**Table 3:** Multi-scale feature extraction based on wavelet transform.

Features	Description
X/Y mutation intensity	The mutation intensity of small-scale energy in the X/Y direction, used to detect collisions and other events
X/Y event count	The number of significant mutation events detected in the X/Y direction
X/Y semi-diurnal tidal energy ratio	The ratio of semi-diurnal tidal energy in the X/Y direction to total energy
X/Y diurnal tidal/energy ratio	The ratio of diurnal tidal energy in the X/Y direction to total energy
X/Y runoff/energy ratio	The ratio of runoff energy in the X/Y direction to total energy
X/Y tidal/runoff ratio	The ratio of tidal-to-runoff energy in the X/Y direction, reflecting the dominant influencing factors
X/Y dominant oscillation type	The dominant movement type in the X/Y direction (semi-diurnal tide/diurnal tide/runoff/mixed)

**Table 4:** Time-domain features extracted from multiple segments.

	Buoy	S1#3black	S2#124black	S3#104red	S4#80red	S5#68black	S6#FN2black	S7#44-1	S8#23black	S9#B5red
Features										
$x_{0}$		399,451.60	414,891.00	457,496.00	490,163.30	508,001.80	541,741.90	28.33	584,817.90	617,268.60
$y_{0}$		3,562,190.00	3,569,676.00	3,565,695.00	3,556,521.00	3,536,871.00	3,543,943.00	32.47	3,527,094.00	3,508,475.00
$\bar{x}$		399,442.60	414,851.00	457,499.50	490,153.40	508,049.40	541,766.20	38.23	584,799.10	617,207.40
$\bar{y}$		3,562,221.00	3,569,634.00	3,565,685.00	3,556,525.00	3,536,885.00	3,543,894.00	3,542,042.00	3,527,076.00	3,508,465.00
$r_{max}$		14.30	26.24	17.11	42.46	44.51	26.52	52.01	44.35	56.25
$\bar{r}$		2.41	5.90	3.29	6.21	7.37	7.71	12.64	15.59	18.60
$\sigma_{r}$		2.26	3.92	2.14	5.62	6.56	5.70	9.44	8.51	10.58
$\sigma_{x}$		2.84	6.72	1.34	4.54	9.17	4.71	14.91	4.66	9.91
$\sigma_{y}$		1.69	2.24	3.68	7.04	3.63	8.35	5.16	17.14	18.97
$R_{xy}$		1.68	3.00	0.36	0.65	2.52	0.56	2.89	0.27	0.52
$S_{x}$		−1.67	−1.16	−0.47	−1.25	−0.34	0.51	−0.65	−0.33	−0.05
$S_{y}$		−0.91	0.08	1.15	1.65	−0.07	−0.51	0.91	0.77	0.72
$K_{x}$		4.20	1.09	0.24	1.59	1.72	0.00	0.27	−0.44	−0.51
$K_{y}$		4.03	−0.08	1.03	3.16	1.27	−0.22	0.51	−0.69	−0.45
$x_{c}$		399,442.60	414,851.00	457,499.50	490,154.30	508,049.40	541,765.60	557,833.80	584,799.00	617,207.30
$y_{c}$		3,562,221.00	3,569,634.00	3,565,685.00	3,556,524.00	3,536,885.00	3,543,895.00	3,542,042.00	3,527,076.00	3,508,463.00
$r_{max,cluster}$		14.31	26.26	17.11	43.97	44.53	27.69	53.63	44.94	58.03
${\bar{r}}_{cluster}$		2.41	5.90	3.29	6.00	7.37	7.77	12.52	15.49	18.37
$q_{0.00}$		0.05	0.24	0.05	0.14	0.10	0.04	0.22	0.09	0.43
$q_{0.25}$		0.80	3.27	1.91	2.34	2.60	2.53	5.91	9.78	10.62
$q_{0.50}$		1.80	5.15	2.89	5.10	5.20	7.28	10.46	14.88	17.11
$q_{0.75}$		3.25	7.42	3.96	7.91	10.41	11.35	14.86	18.33	24.27
$q_{0.90}$		5.14	10.10	5.87	12.33	17.19	15.82	28.33	28.50	33.66
$q_{0.95}$		7.02	13.58	7.72	18.65	19.44	18.84	32.47	34.19	40.65
$q_{0.98}$		9.69	18.53	9.38	24.62	23.26	20.51	38.23	37.95	45.85

**Table 5:** Time-domain features extracted from different areas in S3.

	Buoy	Straight Channel		Curved Channel		Confluences		Bridge Areas	
Features		#99black	#98black	#103-1red	#103black	#105-1black	#105red	#2red	#2black
$x_{0}$		466,521.00	468,039.00	457,582.00	458,551.00	457,670.00	457,387.00	469,675.00	469,015.00
$y_{0}$		3,564,225.00	3,565,441.00	3,563,992.00	3,563,447.00	3,567,981.00	3,566,861.00	3,566,882.00	3,566,784.00
$\bar{x}$		466,552.00	468,060.00	457,599.00	458,547.00	457,674.00	457,426.00	469,653.00	469,036.00
$\bar{y}$		3,564,235.00	3,565,453.00	3,563,982.00	3,563,448.00	3,567,976.00	3,566,839.00	3,566,886.00	3,566,804.00
$r_{max}$		32.20	17.89	14.40	20.54	25.97	19.10	40.82	32.63
$\bar{r}$		5.50	3.76	3.58	3.48	5.29	6.41	5.85	5.79
$\sigma_{r}$		4.75	3.42	2.04	2.89	3.40	3.31	5.02	5.33
$\sigma_{x}$		6.24	3.72	2.85	3.87	5.40	5.13	2.82	5.00
$\sigma_{y}$		3.72	3.46	2.97	2.35	3.22	5.08	7.17	6.08
$R_{xy}$		1.68	1.07	0.96	1.64	1.68	1.01	0.39	0.82
$S_{x}$		−1.40	−0.84	−0.81	−1.32	−1.18	0.59	−0.23	−1.06
$S_{y}$		−0.87	−0.54	0.84	0.27	0.95	0.37	−1.27	−1.06
$K_{x}$		1.91	1.19	0.51	2.08	0.86	−0.25	0.70	0.96
$K_{y}$		1.01	0.85	0.31	0.43	0.17	−0.78	2.08	1.06
$x_{c}$		466,552.00	468,061.00	457,599.00	458,547.00	457,676.00	457,427.00	469,653.00	469,036.40
$y_{c}$		3,564,235.00	3,565,454.00	3,563,982.00	3,563,448.00	3,567,975.00	3,566,838.00	3,566,886.00	3,566,805.00
$r_{max,cluster}$		32.20	18.76	14.40	20.57	27.85	20.12	40.90	33.42
${\bar{r}}_{cluster}$		5.50	3.72	3.58	3.48	5.02	6.43	5.83	5.65
$q_{0.00}$		0.01	0.02	0.04	0.03	0.13	0.16	0.04	0.02
$q_{0.25}$		1.75	1.27	2.21	1.08	3.15	4.24	1.74	1.54
$q_{0.50}$		4.74	2.49	3.29	3.02	4.65	5.87	5.21	4.05
$q_{0.75}$		7.25	5.63	4.37	4.90	6.29	8.27	8.07	8.77
$q_{0.90}$		11.16	9.09	6.11	7.04	9.90	11.53	11.54	13.02
$q_{0.95}$		15.96	11.42	7.60	9.39	12.95	12.19	16.45	17.23
$q_{0.98}$		19.80	12.88	9.33	11.75	15.47	13.04	21.10	20.62

**Table 6:** Wavelet transform features of multi-segments buoys.

	Buoys	S1	S2	S3	S4	S5	S6	S7	S8	S9
Features		#3black	#124black	#104red	#80red	#68black	#FN2black	#44-1	#23black	#B5red
X mutation intensity		5.82	4.36	2.74	3.69	5.47	2.89	3.78	2.75	2.22
X event count		107.00	107.00	107.00	120.00	84.00	119.00	121.00	102.00	122.00
X semi-diurnal energy ratio		2.80	6.16	0.93	2.14	3.42	3.76	4.88	1.59	3.18
X diurnal energy ratio		0.86	1.20	0.61	0.46	0.70	0.55	0.88	0.36	0.38
X runoff/energy ratio		1.01	0.71	1.32	1.39	1.05	1.15	0.91	1.39	1.44
X tidal/runoff ratio		3.63	10.32	1.16	1.87	3.92	3.76	6.31	1.40	2.48
Y mutation intensity		3.72	3.08	4.56	4.72	7.09	2.84	3.47	1.74	2.46
Y event count		89.00	108.00	94.00	128.00	81.00	116.00	128.00	140.00	128.00
Y semi-diurnal energy ratio		0.55	0.61	7.09	5.89	0.98	6.42	2.17	9.65	8.78
Y diurnal energy ratio		0.70	0.47	0.98	1.08	0.57	0.64	0.58	1.29	0.73
Y runoff/energy ratio		1.18	1.62	0.82	0.72	1.47	0.79	1.44	0.60	0.82
Y tidal/runoff ratio		1.06	0.66	9.87	9.69	1.05	8.91	1.91	18.12	11.64
X dominant oscillation type		semi-diurnal	semi-diurnal	runoff	semi-diurnal	semi-diurnal	semi-diurnal	semi-diurnal	semi-diurnal	semi-diurnal
Y dominant oscillation type		runoff	runoff	semi-diurnal	semi-diurnal	runoff	semi-diurnal	semi-diurnal	semi-diurnal	semi-diurnal

## Data Availability

Data are contained within the article.
